# The Variation in the Absence of the Palmaris Longus in a Multiethnic Population of the United States: An Epidemiological Study

**DOI:** 10.1155/2012/282959

**Published:** 2012-10-31

**Authors:** Ali M. Soltani, Mirna Peric, Cameron S. Francis, Thien-Trang J. Nguyen, Linda S. Chan, Alidad Ghiassi, Milan V. Stevanovic, Alex K. Wong

**Affiliations:** ^1^Division of Plastic Surgery, Department of Surgery, Keck School of Medicine, University of Southern California, Los Angeles, CA 90033, USA; ^2^Department of Orthopedic Surgery, Keck School of Medicine, University of Southern California, Los Angeles, CA 90033, USA

## Abstract

The absence of the palmaris longus (PL) has been shown to vary based on body side, gender, and ethnicity. In prior studies, homogenous ethnic populations have been shown to have differences in rates of absence. However, no study thus far has analyzed the differences in palmaris longus prevalence in a multiethnic population. We prospectively collected data on 516 patients visiting the outpatient hand clinics at LAC+USC Medical Center and Keck Medical Center. Analysis of the data was then performed for variables including ethnicity, laterality, and gender. There were no differences in the absence of the PL based on laterality or gender. Ethnically, there was no difference between white (non-Hispanic) and white (Hispanic) patients, with prevalence of 14.9% and 13.1%, respectively. However, African American (4.5%) and Asian (2.9%) patients had significantly fewer absences of the PL than the Caucasian, Hispanic reference group (*P* = 0.005 and *P* = 0.008, resp.). African Americans and Asians have a decreased prevalence of an absent PL. The Caucasian population has a relatively greater prevalence of an absence of the PL. This epidemiological study demonstrates the anatomic variation in this tendon and may be taken into account when planning an operation using tendon grafts.

## 1. Introduction

The palmaris longus (PL) muscle is a slender, superficial flexor muscle of the forearm whose presence is anatomically highly variable and in many cases absent, either unilaterally or bilaterally. The presence of the PL can be determined through noninvasive and standard physical examination of the volar wrist [1, 2]. Several exams have been described which test for the PL, the standard being Schaeffer's test in which the patient joins the thumb to little finger while flexing the wrist [3]. It has been suggested that the palmaris longus contributes to the strength of thumb abduction and may provide an advantage to sports that require hand grip [4, 5]; however, most studies have shown that absence of the PL is not associated with any significant physical or functional deficits, and therefore, the PL is frequently harvested for use in many different hand, reconstructive, and orthopedic surgeries [3, 6–10]. The PL has a characteristically short belly and long tendon, making it an ideal donor for tendon grafts for secondary tendon reconstruction, tendon transfers, and other reconstructive efforts [11].

The absence of the PL has been shown to vary based on body side, gender, and ethnicity in prior studies [1, 12–14]. Interestingly, Erić et al. in 2011 reported on the differential absence of the PL in comparison to hand dominance [1]. They concluded that the PL was more likely to be absent on the nondominant hand [1]. Hereditary variables have been examined in specific racial populations, specifically Nigerian, Caucasian, and Chinese [3, 6, 15–17]. In a Caucasian population, unilateral absence was 16% and bilateral absence was 9%, with males being more affected [2]. In contrast, unilateral and bilateral PL absence is far less common among the Chinese, with 3% and 1%, respectively. All prior anatomical variation studies of the PL have been conducted in separate homogenous populations, such as the Chinese study previously cited. No studies to date have examined the anatomical variation of this tendon in a multiethnic population such as in the United States. Additionally, some studies have shown correlations between PL absence and certain anatomical anomalies, such as an anomalous superficial palmar arch [18].

Taking into account the large variability in the PL absence, 2.8 to 24%, our goal was to examine the prevalence of the PL absence in the multiethnic population of the Los Angeles County + University of Southern California Medical Center (LAC + USC) and the Keck Medical Center of the University of Southern California (USC), reflecting the demographics of Los Angeles [19]. The demographic predictive data could then be used by surgeons worldwide to provide information on possible absence of this valuable tendon preoperatively.

## 2. Methods

**Figure 1:**
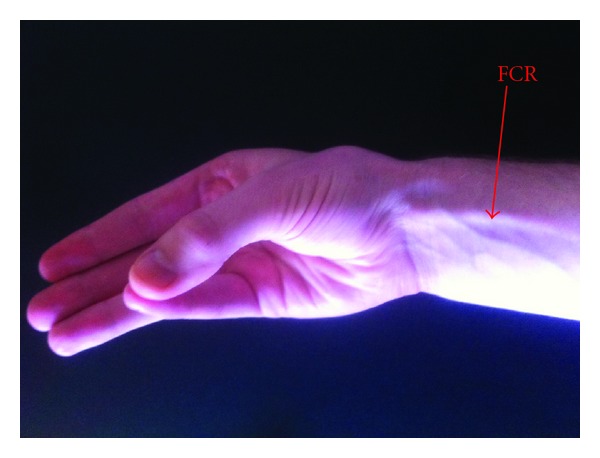
Caucasian patient demonstrating an absent PL.

**Figure 2:**
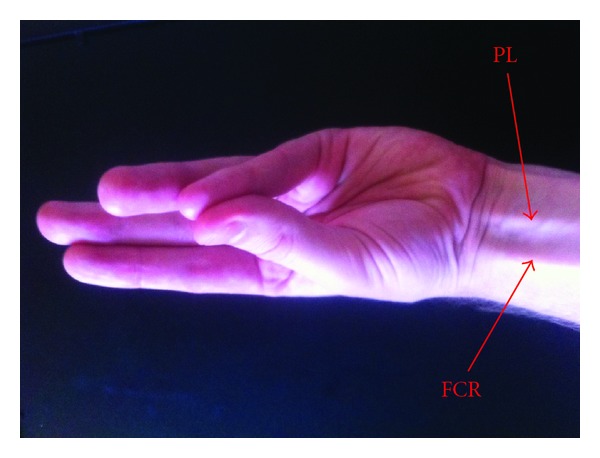
Caucasian patient demonstrating a present PL.

Patients were prospectively followed and evaluated in the hand surgery clinics at the Los Angeles County + University of Southern California Medical Center (LAC + USC) and Keck Medical Center of the University of Southern California (USC). Our objective was to evaluate the extent to which PL is unilaterally and bilaterally absent with regard to age, gender, race, ethnicity, and laterality (right or left). Institutional Review Board (IRB) approval was granted through the University of Southern California (USC) prior to conducting the study. The study size of 500 patients was determined by our statistician to be of sufficient power to prove significance prior to our beginning of the data collection phase of the study. In total, 516 patients were evaluated in this multiethnic population, and statistical data was collected and recorded. Demographic data was provided by an informational sheet, including age, sex, race, and ethnicity. After verifying the self-reported data, the presence or absence of the PL tendon was identified by using the Schaeffer's test and recorded. This examination was performed first by our trained research staff and confirmed by an attending hand surgeon. An example examination is demonstrated in Figures 1 and 2. Ultrasound was available in cases of morbid obesity or difficult diagnosis but was not used in our series of patients in this study. Patients were placed into specific race and ethnic groups based on the United States Census designations. Analysis of the data was then performed for variables including ethnicity, laterality, and gender. Patients with mixed ethnicity or other than Asian, African American, or White were excluded only from the ethnicity portion of the statistical analysis. Statistical analysis of the data using the chi squared test was then performed using Prism version 5 (GraphPad Software, La Jolla, CA) and verified by our statistician (LS Chan).

## 3. Results

The study population included 516 patients in total, including 415 Caucasian, 55 African American, and 35 Asian subjects, and 11 patients of other/mixed origin. Their ages ranged from 12 to 94 years of age, with an average of 42.2 years. There were 288 male patients (55.8%) and 228 female patients (44.2%) evaluated in the hand clinics. There were no differences in the absence of the PL based on laterality. The right side was absent in 11.8% and left 12.0% of the time (see Table 1). Further, there were no differences in the absence of the PL based on gender, *P* value 0.369 (see Table 2). Ethnically, there was no difference in the absence of the PL between White (non-Hispanic) and White (Hispanic) patients, with prevalence of 14.9% and 13.1%, respectively. However, African American (4.5%) and Asian (2.9%) patients had significantly fewer absences of the PL than the Hispanic reference group (*P* = 0.005 and *P* = 0.008, resp.), please see Table 3 for details. 

## 4. Discussion

The palmaris longus has a highly variable prevalence in different ethnic populations. This has been demonstrated priorly in published reports from countries such as Northern Ireland, India, China, Malaysia, and Turkey. These countries have a fairly homogenous ethnic population, in contrast to the United States. Our study population reflects the current multiethnic population of the county of Los Angeles, and hence, is a novel study demonstrating the ethnic variability in the presence of the absent PL. Here, we corroborate the previous Asian studies by showing that our Asian population does, in fact, have a much lower prevalence of absent PL (2.9%). Clinically, for the surgeon, it is a valuable fact that one can be fairly certain for a patient of Asian background that the PL will be highly likely to be present. This is information that should be taken into account preoperatively when planning surgical algorithms in treating tendon injuries or palsy. The PL is one such option as a tendon transfer for opponensplasty in restoring intrinsic function in cases of recurrent median nerve injury. If the PL is absent on the affected side, it is important to know preoperatively to plan using another donor muscle such as the extensor indicis proprius. In our study, the African American population had a statistically significantly lower rate of absent PL (4.5%), which is radically different than previously published reports from Nigeria, where the absence rates were much higher (31%). This could be due to the ethnic heterogeneity of the African American population of the United States compared to the Nigerian population. Nevertheless, the PL is present in high likelihood in this particular ethnic group which bodes well for using the PL in a surgical scenario. The PL is used quite frequently in cases of secondary tendon reconstruction, and it is useful for the surgeon to be aware of that issue preoperatively for surgical safety and efficiency in harvesting the tendon graft. The patient needs to be aware of the location of possible surgical incisions for tendon harvesting. Further, the surgeon should examine all possible tendon donors preoperatively, and one's suspicion might be heightened by knowing the patient's ethnicity. This is particularly important for the White population which in our study had the highest rates of absence, in both the Hispanic and non-Hispanic subsets. The surgeon must be aware in these patients that it is more likely that the PL might be absent. Thus, in Caucasian patients, it is particularly important to have a thorough examination of possible tendon donor sites. However, we did not come to the same conclusion in our study, as the right and left sides were absent in 11.8% and 12.0% of patients, respectively.Our study was limited by the ethnic demographics of the patients visiting the affiliated hand clinics of USC, and the population samples were not evenly distributed between the four groups. The distribution of patients included a majority of Hispanics and a relative scarcity of Asians and African Americans, which could be a source of sampling error due to the low sample size. Nevertheless, statistical significance was confirmed and the data set stayed within the predetermined power guideline. Thus, this epidemiological study demonstrates the ethnically based anatomic variation in this tendon, which has practical clinical application.

## Figures and Tables

**Table 1:** Laterality compared to absence of the PL.

Hand affected	Absent PL	*P*
Right (*n* = 516)	61 (11.8%)	0.924^a^
Left (*n* = 516)	62 (12.0%)	

^a^Chi-squared (*χ* ^2^ = 0.01, df = 1).

**Table 2:** Gender compared to absence of the PL.

Gender	Bilaterally absent PL	Unilaterally absent PL	No absence of PL	*P*
Male (*n* = 288)	19 (6.6%)	22 (7.6%)	247 (85.8%)	0.369^a^
Female (*n* = 228)	20 (8.8%)	23 (10.1%)	185 (81.1%)	

Total (*n* = 516)	39 (7.6%)	45 (8.7%)	432 (83.7%)	

^a^Chi-squared (*χ* ^2^ = 2.00, df = 2).

PL: palmaris longus.

**Table 3:** Ethnicity compared to absence of the PL.

Ethnicity	Absent PL	Odds ratio	95% CI	*P*
White				
Hispanic (*n* = 662)	87 (13.1%)	1.00	Reference group	—
Non-Hispanic (*n* = 168)	25 (14.9%)	1.16	0.71–1.87	0.530
African American (*n* = 110)	5 (4.5%)	0.31	0.12–0.79	**0.005**
Asian (*n* = 70)	2 (2.9%)	0.19	0.05–0.81	**0.008**

Total (*n* = 1010)	119 (11.8%)			

Chi-squared (*χ* ^2^ = 13.64, df = 3, *P* = 0.003).

PL: palmaris longus; CI: confidence interval.
